# Spontaneous pseudoaneurysm of the posterior tibial artery successfully treated by open surgery: Case report and review of the literature

**DOI:** 10.1097/MD.0000000000021523

**Published:** 2020-07-31

**Authors:** Kai Liu, Lin Mu, Shuai Yan, Renshi Ma, Bin Liu

**Affiliations:** a Department of Hand and Foot Surgery; b Department of Radiology; c Department of Operating Room, The First Hospital of Jilin University; d Department of Vascular Surgery, Eastern Division of The First Hospital of Jilin University, China.

**Keywords:** open surgery, posterior tibial artery, pseudoaneurysm, spontaneous

## Abstract

**Rationale:**

An extremely rare spontaneous pseudoaneurysm (PSA) of the posterior tibial artery (PTA) in a middle-aged male patient was cured by open surgery effectively.

**Patient concerns:**

A 53-year-old man presented with the increasing swollen left shank for 1 day, with intermittent pain, pulselessness and pallor. He denied the history of trauma, infection, and other diseases.

**Diagnoses:**

Physical examination, past medical history, ankle brachial index, ultrasonography, computed tomographic angiography (CTA), 3-dimensional reconstruction image of the popliteal artery and its branches and histological examination of intraluminal thrombus and clots helped us diagnose the patient as spontaneous PSA of PTA.

**Interventions:**

Our patient underwent excision of PSA and repair operation of PSA.

**Outcomes:**

The patient recovered well at 2-year follow-up.

**Lessons:**

This rare case provides valuable insights for tissue repair and vascular surgery. Therapeutic methods should be in accordance with the best interest of patient. Open surgery is the effective treatment for spontaneous PSA of PTA.

## 1 Introduction

Pseudoaneurysm (PSA) of the posterior tibial artery (PTA) is uncommon and usually associated with trauma and iatrogenic complication.^[1–3]^ Infection and autoimmune diseases also can be the reasons.^[4]^ A rare type of PSA is spontaneous which occurs in the absence of trauma and other causes. It can be a diagnostic dilemma and a possible cause of limb loss.^[5]^ PSA usually presents with pain, swelling, or an expanding mass. Spontaneous PSA may be asymptomatic and have a subtle delayed presentation.^[6]^ Operative exposure and repair of these injured arteries can be challenging, and surgical managements include direct arterial or patch repair, arterial ligation and graft interposition.^[7]^ Other options including thrombin injection, endovascular intervention and coil embolization have been reported.^[2,8,9]^ Here we describe a rare case that open surgery treatment of a symptomatic spontaneous PSA of PTA and review the current literatures.

## 2 Case report

**Figure 1:**
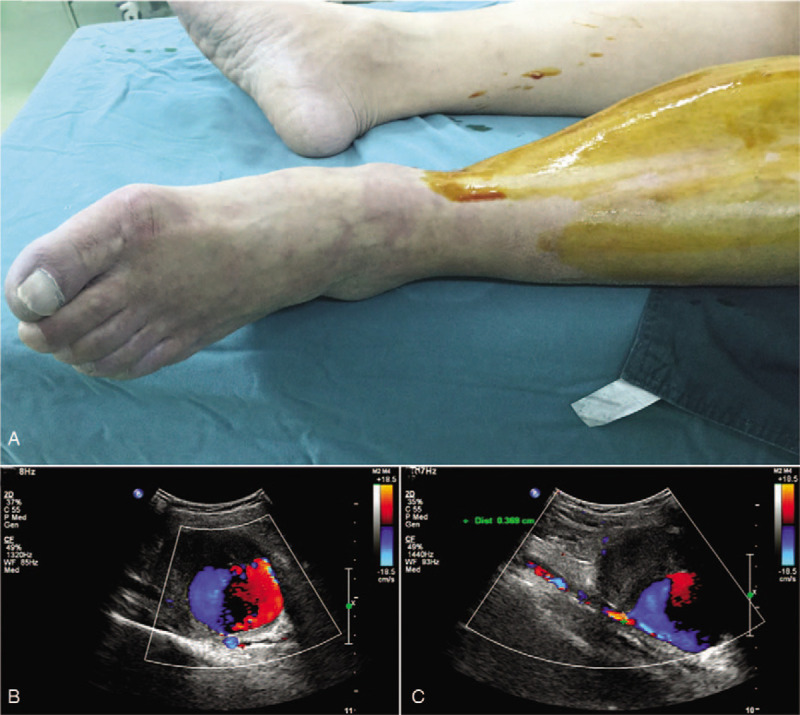
(A) Picture of the preoperative ischemic left shank and foot. (B) Ultrasonographic image of the bloodstream within PSA and (C) the neck and lateral thrombus of PSA. PSA = pseudoaneurysm.

**Figure 2:**
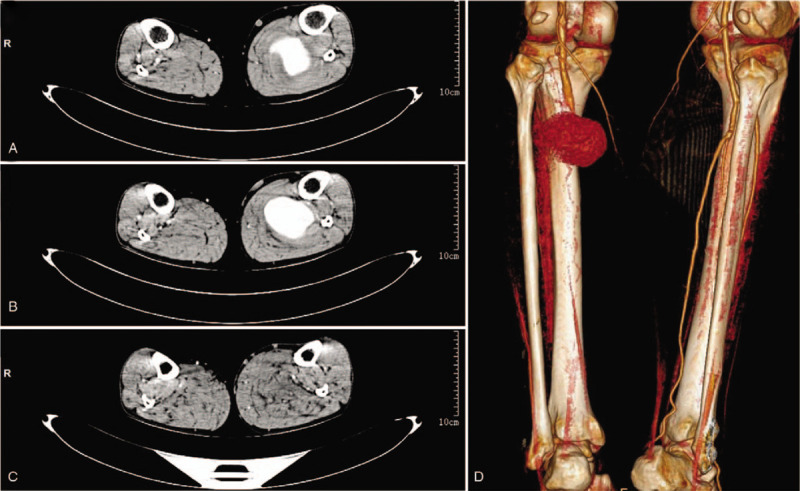
(A) and (B) Axial CTA scans are revealing cross-section of the PSA and (C) the swollen left shank. (D) Three-dimensional reconstruction image demonstrates the volume of PSA. CTA = computed tomographic angiography, PSA = pseudoaneurysm.

A 53-year-old man sought help to emergency department because of the increasing swollen left shank for 1 day, with intermittent pain, pulselessness and pallor. He denied any history of drug abuse, trauma, local infection, intermittent claudication, previous surgery, rheumatic heart disease, atrial fibrillation or infective endocarditis. Blood pressure of our patient was 162/95 mmHg, with no cardiac murmurs and peripheral bruits. Physical examination revealed swelling of the left leg and a palpable pulsatile mass. Pulsation of the dorsalis pedis artery (DPA) and PTA were vanished. Skin temperature of the shank was measured by an infrared thermometer (Bailing Medical Equipment Co. Ltd. China). The cyanotic left toes were clearly visible (Fig. 1A). Blood tests, echocardiography and electrocardiogram (ECG) of our patient were normal. Ankle-brachial index (ABI) of our patient measured by Vista AVS (Summit Doppler Systems, Inc. USA) was abnormal. Ultrasound images showed a PSA with a volume of 79 mm × 62 mm and a neck diameter of 3.69 mm (Fig. 1B and C). Computed tomographic angiography (CTA) images revealed a 61.7 mm × 72 mm PSA of the left shank (Fig. 2A and B). Soft tissue dropsy of the left shank was clearly visible in the image (Fig. 2C). Three-dimensional reconstruction image showed the volume of PSA and disruption of PTA (Fig. 2D). PSA was so large that it compressed muscles of the calf and some of main arteries of the calf. Therefore, we selected open surgery to remedy our patient.

**Figure 3:**
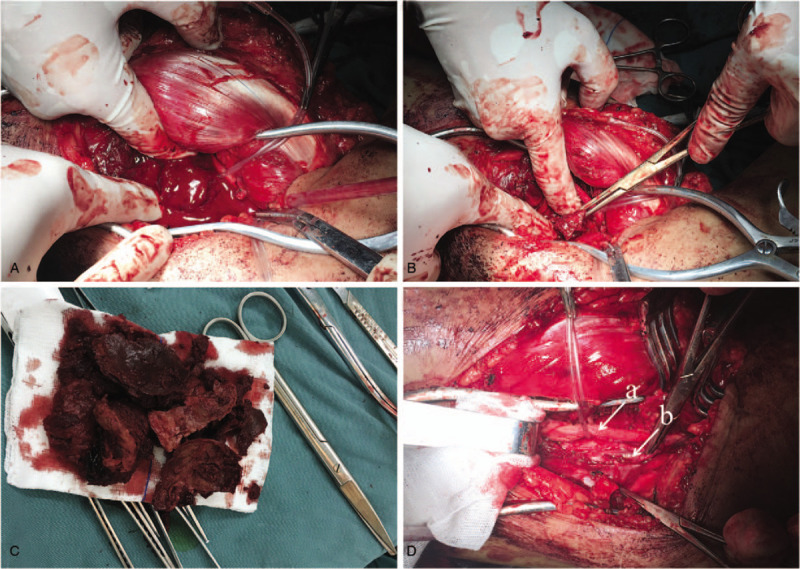
(A) and (B) Intraoperative photos demonstrate impaling PSA and evacuating clots and thrombus. (C) Intraluminal thrombus of PSA. (D) Intraoperative view of PSA resected and perforation of PTA exposed (a = tibial nerve, b = PTA). PSA = pseudoaneurysm, PTA = posterior tibial artery.

**Figure 4:**
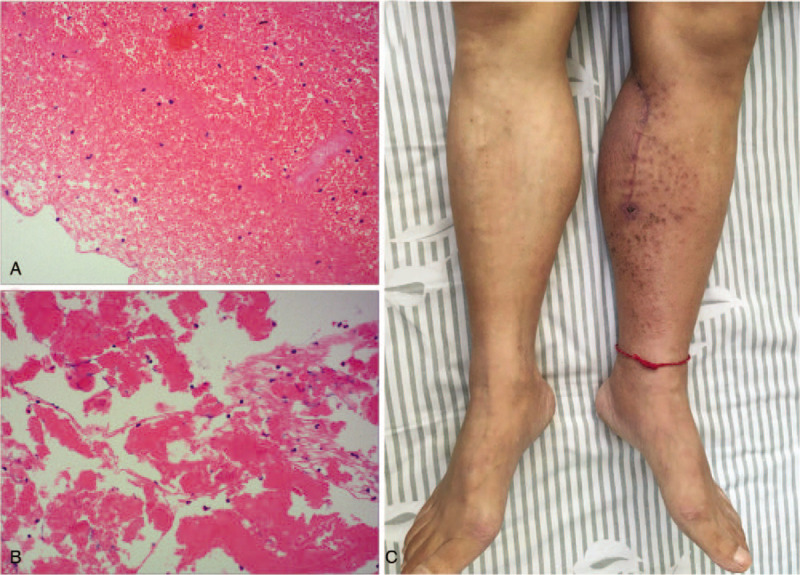
(A) and (B) Histological examination confirmed the intraluminal thrombus of PSA without any evidence of disorders of connective tissue, necrotizing vasculitis and arteritis. (C) Bilateral shanks and feet at 6-month follow-up. PSA = pseudoaneurysm.

Blunt dissection of calf muscle groups was operated during the operation. PSA was punctured to relieve the pressure of the left calf (Fig. 3 A). A mass of intraluminal thrombus and clots were removed (Fig. 3B and C). A perforation was found in PTA which was repaired with 6–0 blood vessel sutures after the tibial nerve was protected (Fig. 3D). Pulsation of PTA was recovered well after the operation. Histological examination confirmed intraluminal thrombus of PSA with inflammatory cells, without any evidence of disorders of connective tissue, necrotizing vasculitis and arteritis (Fig. 4 A and B). Skin temperature and ABI of the left shank after surgery were significantly improved compared with those before surgery. Antibiotic therapy (Ceftezole Sodium) was appropriately given for 7 days after surgery. He was discharged 9 days after surgery with no complication.

**Figure 5:**
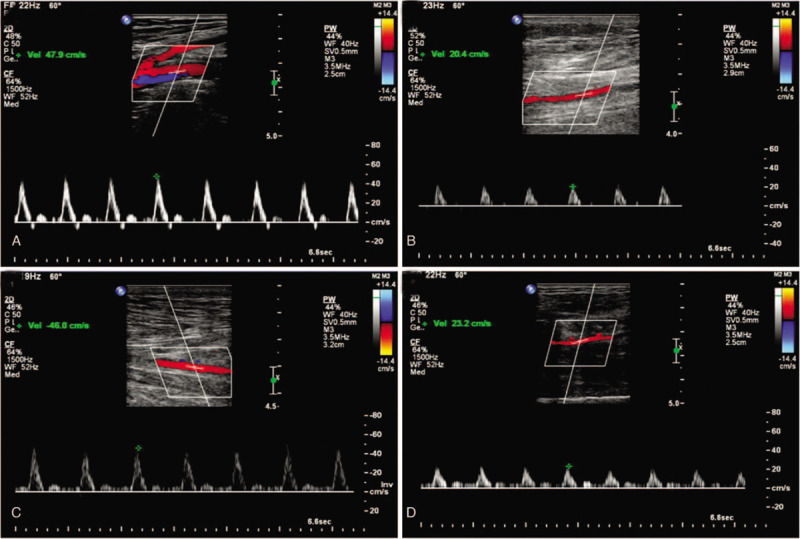
Six-month follow-up ultrasonographic images of popliteal artery (A), ATA (B), PTA (C) and peroneal artery (D). ATA = anterior tibial artery, PTA = posterior tibial artery.

Regular activity of our patient came back a few weeks after discharge. Walking distance of the patient was undiminished. At 6-month follow-up visit, we found the left lower extremity showed a well-healing surgical incision (Fig. 4C). Pulsations of left PTA and DPA were easily palpable. Skin temperatures of the left shank and foot were equal to the opposite side (Table 1). ABI of the left lower extremity also improved significantly compared with the previous test (Table 2). Ultrasound images showed that the blood supply of popliteal artery and its branches in the left lower extremity recovered well (Fig. 5). Our patient had no recurrence of PSA and no clinical symptom at 2-year follow-up.

**Table 1:**
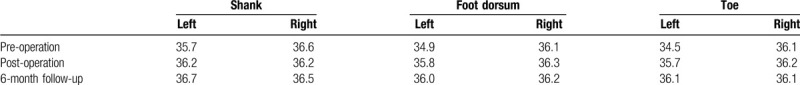
Skin temperature of lower extremity (degrees centigrade).

**Table 2:**
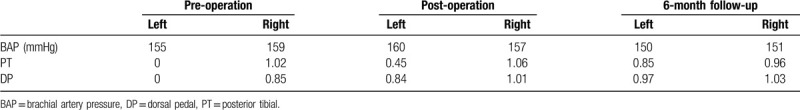
Ankle brachial index.

## 3 Discussion

PSA is localized arterial damage with or without adventitial covering.^[10]^ It is characteristic that local damage of all three arterial layers leading to blood extravasation. The most common site for PSA of lower limb is anterior tibial artery (ATA).^[11]^ PSA of PTA is still rare. Sometimes PSA have resulted from direct penetration of PTA, which allowed for persistent extravasation of blood into the surrounding soft tissues. Extravasation of blood is walled off by circumambient layers of the connective tissues but maintains its connection with the arterial lumen by a neck.^[12]^

Etiologies of PSA are often traumatic,^[13]^ iatrogenic^[14]^ and inflammatory origins.^[15]^ PSA after trauma is becoming a common occurrence because of civilian violence in society increasingly.^[16]^ Other factors include Behcet disease, hemophilia, osteogenesis imperfect, Ehlers-Danlos syndrome, Type IV fibromuscular dysplasia, polyarteritis nodosa and Marfan disease.^[17–19]^ Furthermore, some other factors such as immunosuppression, malnutrition and diabetes are also linked to the risk of PSA formation.^[20]^ Timeframe for detection of PSA is quite fickle and ranges from some days to 5 years.^[21,22]^ A further potential cause of spontaneous PSA of PTA may be prominent and sharp hardware causing attrition damage to structures of arteries. This viewpoint may expound the fickle timeframe for detection of PSA.^[7]^ In our case, although no history of trauma was obtained, our patient was a driver. The possibility could not be eliminated as PSA of PTA asymptomatically developed in unnoticed injury.

Spontaneous PSA of PTA was extremely rare that we could find only four reports in the English literature (Table 3).^[6,23–25]^ Rowe et al in 1987 were first to report a spontaneous PSA of PTA due to an unknown cause that was treated by excision.^[23]^ Our patient had unexplained lesion of PTA, leading to the formation of a spontaneous non-traumatic PSA. Although our patient had hypertension and smoking history, he did not meet the criteria for diagnosis of rheumatic heart disease, atrial fibrillation or infective endocarditis. Preoperative CTA and three-dimensional reconstruction images showed no criteria for diagnosis of arteriosclerosis obliterans. He had no history of intermittent claudication either. The possibility of bacteremia infection was ruled out by preoperative negative blood tests. One etiology of spontaneous PSA is connective tissue disorders. But our patient did not meet the diagnostic criteria of Marfan syndrome, type-IV Ehlers-Danlos syndrome or any other connective tissue disorders.^[4]^ Histological examination of intraluminal thrombus did not suggest any evidence of connective tissue disorders or other specific diseases.

**Table 3:**
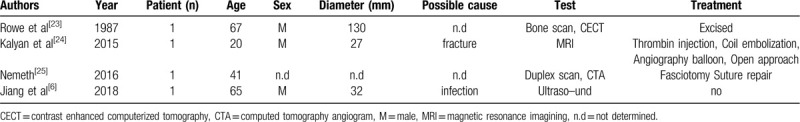
Summary of reported cases of the spontaneous posterior tibial artery pseudoaneurysm.

The clinical manifestation of the PSA is in the form of a throbbing lump. Some small PSA can be often asymptomatic and regress completely when thrombosis occurs.^[26]^ However, if PSA can progressively increase in size, adjacent arteries, veins and peripheral nerves will be compacted. Therefore, pain, swelling, paresthesia and neuralgia appear constantly.^[24]^ Some cases reported symptoms of lower extremity ischemia following PSA thrombosis and distal embolization.^[27,28]^ Early recognition and diagnosis are indispensable to provide prompt clinical treatment. Ultrasonography is usually sufficient for initial diagnosis of PSA.^[18,20]^ CTA and magnetic resonance imagining (MRI) may be more helpful for further diagnosis of this disease and accurate in pinpointing the location and size of PSA.^[29]^ Observation and intervention should be undertaken once the diagnosis of spontaneous PSA is established to prevent enlargement and potential complications sometimes.^[13]^

There is no consensus on overall treatment strategy of PSA currently. The choice of treatment is influenced by the size and location of PSA. It also depends on clinical presentation of PSA, patient's physical condition and comorbidities.^[30]^ Small asymptomatic PSA can be observed for spontaneous resolution. But larger symptomatic masses require to be remedied. At present, endovascular intervention is undertaken as the preferred method to treat peripheral arterial diseases over open surgical treatment. Traditional surgical treatment is associated with complications like bleeding, hematoma and arteriovenous fistula.^[31]^ But traditional surgical treatment remains an important treatment selection for a minority of patients and is reserved for emergent conditions such as rupture PSA, gigantic PSA or other treatments are ineffective. In addition, spontaneous or infection-related PSA are almost always repaired in this way.^[32]^ Furthermore, decompression of the calf soft tissue was required for our patient. Compared with other therapies, such as thrombin injection and endovascular therapy (covered stent or coil), open surgery treatment could decompress the left shank rapidly in our patient.^[17,33]^ For our patient, open approach was the preferred method of remedy with either arterial repair or ligation. Covered stent placement was not an excellent choice because caliber of the artery was too small.^[22]^ Because our patient had blood circulation disorder and soft tissue swelling of the left shank, thrombin injection was not preferable due to presumed aggravation of circulation. The patient benefited from a conventional open surgery treatment, which allowed extensive exploration to repair PTA meticulously. Vascular repair and reconstruction usually involve an end-to-end anastomosis, while venous autograft is usually involved.

Although in most reports we can find a pulsatile mass, we should know that the mass may be pulseless which can cause misdiagnosis and delayed diagnosis.^[34]^ We believe that clinicians should rely on CTA or MRI to diagnose the disease in this condition. The choice of open surgery or endovascular treatment should be in the best interest of the patient.

## Author contributions

**Conceptualization:** Bin Liu.

**Investigation**: Lin Mu

**Data curation:** Shuai Yan.

**Formal analysis:** Kai Liu.

**Resources:** Renshi Ma

**Writing – original draft:** Kai Liu.

**Writing – review & editing:** Bin Liu.
